# Severe obesity in children: prevalence, persistence and relation to hypertension

**DOI:** 10.1186/1687-9856-2014-3

**Published:** 2014-03-03

**Authors:** Joan C Lo, Malini Chandra, Alan Sinaiko, Stephen R Daniels, Ronald J Prineas, Benjamin Maring, Emily D Parker, Nancy E Sherwood, Matthew F Daley, Elyse O Kharbanda, Kenneth F Adams, David J Magid, Patrick J O’Connor, Louise C Greenspan

**Affiliations:** 1 Division of Research, Kaiser Permanente Northern California, 2000 Broadway, Oakland, CA 94612, USA; 2 Department of Medicine, Kaiser Permanente Oakland Medical Center, Oakland, CA, USA; 3 Department of Pediatrics, University of Minnesota, Minneapolis, MN, USA; 4 University of Colorado Denver School of Medicine, Denver, CO, USA; 5 Division of Public Health Sciences, Wake Forest University School of Medicine, Winston-Salem, NC, USA; 6 HealthPartners Institute for Education and Research, Minneapolis, MN, USA; 7 Institute for Health Research, Kaiser Permanente Colorado, Denver, CO, USA; 8 Department of Pediatrics, Kaiser Permanente San Francisco Medical Center, San Francisco, CA, USA

**Keywords:** Obesity, Children, Adolescents, Blood pressure

## Abstract

**Background:**

Newer approaches for classifying gradations of pediatric obesity by level of body mass index (BMI) percentage above the 95^th^ percentile have recently been recommended in the management and tracking of obese children. Examining the prevalence and persistence of severe obesity using such methods along with the associations with other cardiovascular risk factors such as hypertension is important for characterizing the clinical significance of severe obesity classification methods.

**Methods:**

This retrospective study was conducted in an integrated healthcare delivery system to characterize obesity and obesity severity in children and adolescents by level of body mass index (BMI) percentage above the 95^th^ BMI percentile, to examine tracking of obesity status over 2–3 years, and to examine associations with blood pressure. Moderate obesity was defined by BMI 100-119% of the 95^th^ percentile and severe obesity by BMI ≥120% × 95^th^ percentile. Hypertension was defined by 3 consecutive blood pressures ≥95^th^ percentile (for age, sex and height) on separate days and was examined in association with obesity severity.

**Results:**

Among 117,618 children aged 6–17 years with measured blood pressure and BMI at a well-child visit during 2007–2010, the prevalence of obesity was 17.9% overall and was highest among Hispanics (28.9%) and blacks (20.5%) for boys, and blacks (23.3%) and Hispanics (21.5%) for girls. Severe obesity prevalence was 5.6% overall and was highest in 12–17 year old Hispanic boys (10.6%) and black girls (9.5%). Subsequent BMI obtained 2–3 years later also demonstrated strong tracking of severe obesity. Stratification of BMI by percentage above the 95^th^ BMI percentile was associated with a graded increase in the risk of hypertension, with severe obesity contributing to a 2.7-fold greater odds of hypertension compared to moderate obesity.

**Conclusion:**

Severe obesity was found in 5.6% of this community-based pediatric population, varied by gender and race/ethnicity (highest among Hispanics and blacks) and showed strong evidence for persistence over several years. Increasing gradation of obesity was associated with higher risk for hypertension, with a nearly three-fold increased risk when comparing severe to moderate obesity, underscoring the heightened health risk associated with severe obesity in children and adolescents.

## Background

Data from U.S. population surveys demonstrate a significant increase in obesity prevalence among children age 2–19 years old, from 5.5% in 1976–1980 [1] to 16.9% in 2007–2010 [1,2], with obesity defined as body mass index (BMI) ≥95^th^ percentile using the Centers for Disease Control and Prevention (CDC) 2000 growth charts [3]. As obesity rates have climbed in all age groups [4-7], the prevalence of severe obesity has also risen, increasing from 1.1% to 5.1% in boys and 1.3% to 4.7% in girls from 1976–2006 [7]. Historically, severe obesity in children has been described in broad terms, with fewer studies examining gradations of obesity severity in relation to potentially adverse secondary complications. Methods for classifying extremely high BMI have evolved in the past decade, related in part to the limited utility of BMI percentiles and Z scores where contraction of values occurs at the upper range [8]. As an alternative, expressing BMI as a percentage of the 95^th^ BMI percentile has been recommended for characterizing and tracking children with high BMI [9-11], where a threshold of BMI ≥120% of the 95^th^ percentile has been used to define severe obesity [7,9-12]. New growth charts with additional growth curves representing higher order BMI as a percentage of the 95^th^ percentile have also been recently published [10,11] and may allow for more precise stratification of risk among obese children.

The present study conducted in a contemporary, diverse population of children followed in routine pediatric clinical care settings was designed with three specific aims. First, we characterized obesity severity by expressing BMI as a percentage of the 95^th^ BMI percentile for age and sex. Second, we examined obesity status over 2–3 years follow-up, to determine tracking of both obesity and severe obesity in children and early adolescents. Third, we applied these new obesity stratification criteria to examine the association of obesity severity and hypertension as a potential indicator of the degree of cardiovascular health risk.

## Methods

The Institutional Review Board at HealthPartners Institute for Education and Research approved the study with ceding of oversight authority by the KPNC Institutional Review Board. A waiver of informed consent was obtained due to the nature of the study.

Kaiser Permanente Northern California (KPNC) is a large integrated healthcare delivery system providing comprehensive care for more than three million members annually. For this study, we examined data from a large retrospective multicenter study of pediatric hypertension [13], using a subcohort that included 117,936 children aged 6–17 years receiving care within three large KPNC subregions. Data pertaining to age, height, weight and blood pressure were obtained from the electronic medical record for the first (index) well child visit with measured height, weight and blood pressure between July 1, 2007 and December 31, 2010 as previously described [13]. Data for race/ethnicity, sex and membership were obtained from administrative databases.

Height was measured by stadiometer and weight was measured on a calibrated scale as part of routine clinical care. Body mass index (BMI) was calculated as weight in kilograms divided by height in meters squared (kg/m^2^), with percentiles calculated from the year 2000 CDC growth charts and reference datasets [14] to classify children as normal weight or underweight (BMI <85^th^ BMI-for-age percentile); overweight (BMI 85^th^-94^th^ (94.9) BMI-for-age percentile); or obese (BMI ≥95^th^ BMI-for-age percentile) [3,4] as previously described [13,15]. For the purposes of this study, obese children were further classified based on BMI expressed as a percentage above the 95^th^ BMI percentile according to age and sex [9,11], with a BMI 100-119% of the 95^th^ percentile used to define moderate obesity and a BMI ≥120% of the 95^th^ BMI percentile used to define severe obesity [7,9,12]. For children aged 6–13 years, a follow-up BMI was also examined when the child was 2–3 years older (available in 73.1% of subjects, requiring a minimum of 600 days between visits; 89.5% of follow-up visits were for well child care).

To address potential BMI errors in the electronic medical record for the index visit, we first excluded those with height flagged as “biologically implausible” by the CDC program [16] and those with height <91.4 or >213.4 cm, weight <9.1 or >272.2 kg, or BMI <10 or >120 kg/m^2^. Next, 540 growth charts were manually reviewed if the index anthropometric data met any of the following criteria: (1) high values of weight or BMI flagged as “biologically implausible” by the CDC program [16] (except adolescents 12–17 years old with another BMI <5% different from the index value); (2) severely obese children with another height, weight or BMI differing by >15-20% per year within 2 years; and (3) obese children with subsequent non-obese BMI. Review of these outlying values resulted in the exclusion of 97 children with data errors.

Blood pressure measurements were obtained using oscillometric devices as previously described [13], with levels classified according to the Fourth Report on the Diagnosis, Evaluation and Treatment of High Blood Pressure in Children and Adolescents [17]. Normal blood pressure was defined as a systolic and diastolic blood pressure <90^th^ percentile, prehypertension was defined as a systolic or diastolic blood pressure between the 90^th^ and <95^th^ percentile (or blood pressure ≥120/80 mmHg for adolescents) and an elevated BP in the hypertension range was defined by a systolic or diastolic BP ≥95^th^ percentile [17]. Among children and adolescents with an index (initial) elevated BP ≥95^th^ percentile, hypertension was classified based on two additional consecutive BP measurements ≥95^th^ percentile [17] or SBP ≥140 and/or DBP ≥90 mmHg for subsequent BP obtained at age ≥18 years and older (0.24% of the overall cohort) as previously described [13]. There were 730 (3.5% of the overall cohort) who did not have follow-up BP to allow final BP classification.

### Statistical analysis

All analyses were conducted using SAS version 9.3 (SAS Institute, Cary, NC). Means with standard deviation and medians with interquartile range for continuous variables and proportions for categorical variables were computed. Differences between subgroups and classification methods were compared using the chi-square test for categorical variables and Student’s t-test for continuous variables. The trend in proportions across categories was examined using the Cochrane-Armitage test. Multivariable logistic regression was used to examine the independent relationship of obesity severity and elevated blood pressure, adjusting for differences in age, race/ethnicity and sex. These multivariable analyses, conducted in obese children, excluded 107 (0.5%) who received treatment with blood pressure lowering medication (the majority received clonidine or guanfacine, for presumably non-hypertensive indications) in the six months prior to the index well-child visit. A p-value of <0.05 was chosen as the criterion for statistical significance.

## Results

**Figure 1:**
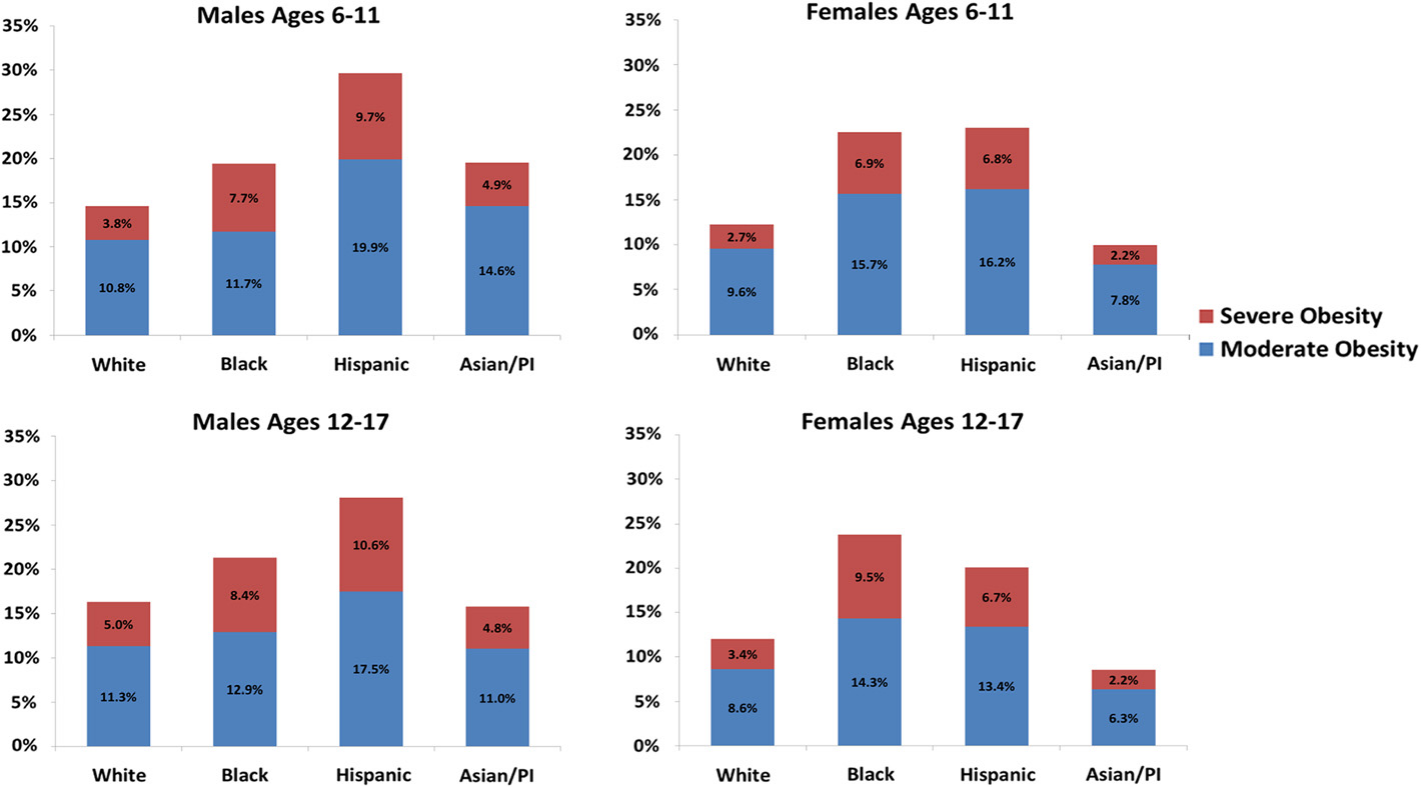
**The proportion of children and adolescents with moderate and severe obesity by age, sex and race/ethnicity.** Comparisons by race/ethnicity within each age-sex group showed that the prevalence of obesity (BMI ≥95^th^ percentile) was significantly different between all racial/ethnic subgroups (p < 0.01) except for differences in obesity prevalence for black vs Asian 6–11 year old boys (p = 0.90), black vs. Hispanic 6–11 year old girls (p = 0.64), and white vs. Asian 12–17 year old boys (p = 0.37). The prevalence of severe obesity (BMI ≥120% of the 95^th^ percentile) was also significantly different between all racial/ethnic subgroups (p < 0.01), except for white vs. Asian (p = 0.06) and black vs. Hispanic 6–11 year old girls (p = 0.96), and white vs. Asian 12–17 year old boys (p = 0.58).

The final study population consisted of 117,618 children and adolescents aged 6–17 years with measured height, weight and blood pressure at well child visits between July 1, 2007 and December 31, 2010. Half (49.5%) were female and 55.3% were age 12–17 years old. There was substantial racial and ethnic diversity, with 31.3% white, 8.8% black, 26.2% Hispanic, 21.0% Asian/Pacific Islander (PI) and the remainder of other or unknown race/ethnicity (12.8%). Overall, 17.9% met criteria for obesity defined by BMI ≥95^th^ percentile, with a greater proportion among boys versus girls, and slightly greater among younger versus older age group (Table 1). Differences were also seen by race/ethnicity. Among boys, the highest prevalence of obesity was seen in Hispanics (28.9%), followed by blacks (20.5%), Asians (17.6%) and whites (15.6%, p < 0.001 for all comparisons). Among girls, the highest prevalence was seen in blacks (23.3%) followed by Hispanics (21.5%), whites (12.1%) and Asians (9.2%, p <0.01 for all comparisons). Figure 1 further compares prevalence findings by race/ethnicity within each age-sex subgroup. Overall, 5.6% of the cohort had severe obesity defined by BMI ≥120% of the 95^th^ BMI percentile, with a greater proportion among boys compared to girls (6.6% vs. 4.5%, p < 0.001). The prevalence of severe obesity also varied by race/ethnicity, ranging from 3.5% in Asians and 3.8% in whites to 8.3% in blacks and 8.5% in Hispanics (p <0.001 for Asians and whites vs. blacks and Hispanics). When examined across age-sex subgroups (Figure 1), the prevalence of severe obesity was highest in Hispanic boys aged 6–11 (9.7%) and 12–17 (10.6%) years and Black girls aged 12–17 (9.5%) years. In multivariable analyses, increasing age, male sex (adjusted odds ratio OR 1.5, 95% confidence interval CI 1.5-1.6), Hispanic ethnicity (OR 2.4, 95% CI 2.2-2.5) and black race (OR 2.3, 95% CI 2.1-2.5) were independent predictors of severe obesity.

**Table 1:** Body mass index (BMI) classification by BMI percentile for children age 6–17 years old

		**BMI < 5**^**th **^**percentile**	**BMI 5-84**^**th **^**percentile**	**BMI 85-94**^**th **^**percentile**	**BMI ≥ 95**^**th **^**percentile**	**BMI ≥ 120% times the 95**^**th **^**percentile**
**OVERALL**	**117618**	**2679 (2.3%)**	**73630 (62.6%)**	**20296 (17.3%)**	**21013 (17.9%)**	**6532 (5.6%)**
Race/ethnicity						
White	36793	892 (2.4%)	24859 (67.6%)	5941 (16.2%)	5101 (13.9%)	1401 (3.8%)
Black	10306	150 (1.5%)	5921 (57.5%)	1980 (19.2%)	2255 (21.9%)	853 (8.3%)
Hispanic	30821	447 (1.5%)	16609 (53.9%)	6001 (19.5%)	7764 (25.2%)	2618 (8.5%)
Asian/PI	24701	828 (3.4%)	16753 (67.8%)	3789 (15.3%)	3331 (13.5%)	873 (3.5%)
Other/unkn	14997	362 (2.4%)	9488 (63.3%)	2585 (17.2%)	2562 (17.1%)	787 (5.3%)
Age group						
6 – 11 yrs	52567	1325 (2.5%)	32619 (62.1%)	8897 (16.9%)	9726 (18.5%)	2773 (5.3%)
12 – 17 yrs	65051	1354 (2.1%)	41011 (63.0%)	11399 (17.5%)	11287 (17.4%)	3759 (5.8%)
**BOYS**	**59380**	**1432 (2.4%)**	**35870 (60.4%)**	**9924 (16.7%)**	**12154 (20.5%)**	**3935 (6.6%)**
Race/ethnicity						
White	18488	503 (2.7%)	12262 (66.3%)	2843 (15.4%)	2880 (15.6%)	830 (4.5%)
Black	5195	88 (1.7%)	3140 (60.4%)	902 (17.4%)	1065 (20.5%)	421 (8.1%)
Hispanic	15507	230 (1.5%)	7983 (51.5%)	2819 (18.2%)	4475 (28.9%)	1580 (10.2%)
Asian/PI	12661	417 (3.3%)	7967 (62.9%)	2049 (16.2%)	2228 (17.6%)	613 (4.8%)
Other/unkn	7529	194 (2.6%)	4518 (60.0%)	1311 (17.4%)	1506 (20.0%)	491 (6.5%)
Age group						
6 – 11 yrs	26977	633 (2.4%)	16075 (59.6%)	4607 (17.1%)	5662 (21.0%)	1702 (6.3%)
12 – 17 yrs	32403	799 (2.5%)	19795 (61.1%)	5317 (16.4%)	6492 (20.0%)	2233 (6.9%)
**GIRLS**	**58238**	**1247 (2.1%)**	**37760 (64.8%)**	**10372 (17.8%)**	**8859 (15.2%)**	**2597 (4.5%)**
Race/ethnicity						
White	18305	389 (2.1%)	12597 (68.8%)	3098 (16.9%)	2221 (12.1%)	571 (3.1%)
Black	5111	62 (1.2%)	2781 (54.4%)	1078 (21.1%)	1190 (23.3%)	432 (8.5%)
Hispanic	15314	217 (1.4%)	8626 (56.3%)	3182 (20.8%)	3289 (21.5%)	1038 (6.8%)
Asian/PI	12040	411 (3.4%)	8786 (73.0%)	1740 (14.5%)	1103 (9.2%)	260 (2.2%)
Other/unkn	7468	168 (2.3%)	4970 (66.6%)	1274 (17.1%)	1056 (14.1%)	296 (4.0%)
Age group						
6 – 11 yrs	25590	692 (2.7%)	16544 (64.7%)	4290 (16.8%)	4064 (15.9%)	1071 (4.2%)
12 – 17 yrs	32648	555 (1.7%)	21216 (65.0%)	6082 (18.6%)	4795 (14.7%)	1526 (4.7%)

Numbers represent row percentages; yrs= years; unkn= unknown.

Table 2 shows the proportion of children within each BMI percentage category above the 95^th^ percentile, by age and sex. For the 21,013 obese children (BMI ≥95^th^ percentile), nearly one third were severely obese (BMI ≥120% of the 95^th^ percentile). Half of severely obese youth had BMI 120-129% of the 95^th^ percentile and the remainder were evenly distributed across BMI 130-139% and BMI ≥140% of the 95^th^ percentile. The remaining two thirds of obese children met criteria for moderate obesity (BMI 100-119% of the 95^th^ percentile). For both boys and girls with BMI ≥95^th^ percentile, a greater proportion of older compared to younger children met criteria for severe obesity (p < 0.05 comparing age 6–11 vs. 12–17 years old).

**Table 2:** **Characteristics of obese children and adolescents based on percentage of the 95**^**th **^**percentile for body mass index (BMI)**

	**Overall N = 21,013**	**100-109% × 95**^**th **^**percentile**	**110-119% × 95**^**th **^**percentile**	**≥ 120% × 95**^**th **^**percentile**	**120-129% × 95**^**th **^**percentile**	**130-139% × 95**^**th **^**percentile**	**≥ 140% × 95**^**th **^**percentile**
**OVERALL**	**21,013**	**8923 (42.5%)**	**5558 (26.5%)**	**6532 (31.1%)**	**3172 (15.1%)**	**1701 (8.1%)**	**1659 (7.9%)**
Age group							
6 – 11 yrs	9726	4314 (44.4%)	2639 (27.1%)	2773 (28.5%)	1486 (15.3%)	702 (7.2%)	585 (6.0%)
12 – 17 yrs	11287	4609 (40.8%)	2919 (25.9%)	3759 (33.3%)	1686 (14.9%)	999 (8.9%)	1074 (9.5%)
BMI, mean ± SD							
Age 6 – 11 yrs	25.1 ± 3.7	22.7 ± 2.0	25.0 ± 2.1	29.0 ± 3.7	27.1 ± 2.2	29.4 ± 2.5	33.4 ± 4.2
Age 12 – 17 yrs	31.9 ± 4.9	28.4 ± 1.7	31.0 ± 1.9	36.9 ± 4.8	33.7 ± 2.0	36.5 ± 2.1	42.3 ± 5.0
**BOYS**	**12154**	**5032 (41.4%)**	**3187 (26.2%)**	**3935 (32.4%)**	**1946 (16.0%)**	**1015 (8.4%)**	**974 (8.0%)**
Age group							
6 – 11 yrs	5662	2449 (43.3%)	1511 (26.7%)	1702 (30.1%)	910 (16.1%)	426 (7.5%)	366 (6.5%)
12 – 17 yrs	6492	2583 (39.8%)	1676 (25.8%)	2233 (34.4%)	1036 (16.0%)	589 (9.1%)	608 (9.4%)
BMI, mean ± SD							
Age 6 – 11 yrs	24.9 ± 3.6	22.4 ± 1.9	24.7 ± 2.0	28.6 ± 3.6	26.7 ± 2.1	29.0 ± 2.3	32.7 ± 4.1
Age 12 – 17 yrs	31.4 ± 4.6	27.8 ± 1.5	30.4 ± 1.7	36.1 ± 4.5	33.2 ± 1.8	35.9 ± 2.0	41.5 ± 4.5
**GIRLS**	**8859**	**3891 (43.9%)**	**2371 (26.8%)**	**2597 (29.3%)**	**1226 (13.8%)**	**686 (7.7%)**	**685 (7.7%)**
Age group							
6 – 11 yrs	4064	1865 (45.9%)	1128 (27.8%)	1071 (26.4%)	576 (14.2%)	276 (6.8%)	219 (5.4%)
12 – 17 yrs	4795	2026 (42.3%)	1243 (25.9%)	1526 (31.8%)	650 (13.6%)	410 (8.6%)	466 (9.7%)
BMI, mean ± SD							
Age 6 – 11 yrs	25.5 ± 3.8	23.1 ± 2.1	25.5 ± 2.2	29.7 ± 3.9	27.7 ± 2.4	30.1 ± 2.6	34.4 ± 4.2
Age 12 – 17 yrs	32.6 ± 5.0	29.0 ± 1.7	31.8 ± 1.9	38. ± 5.1	34.7 ± 1.9	37.4 ± 2.1	43.4 ± 5.5

Numbers represent row percentages.

**Figure 2:**
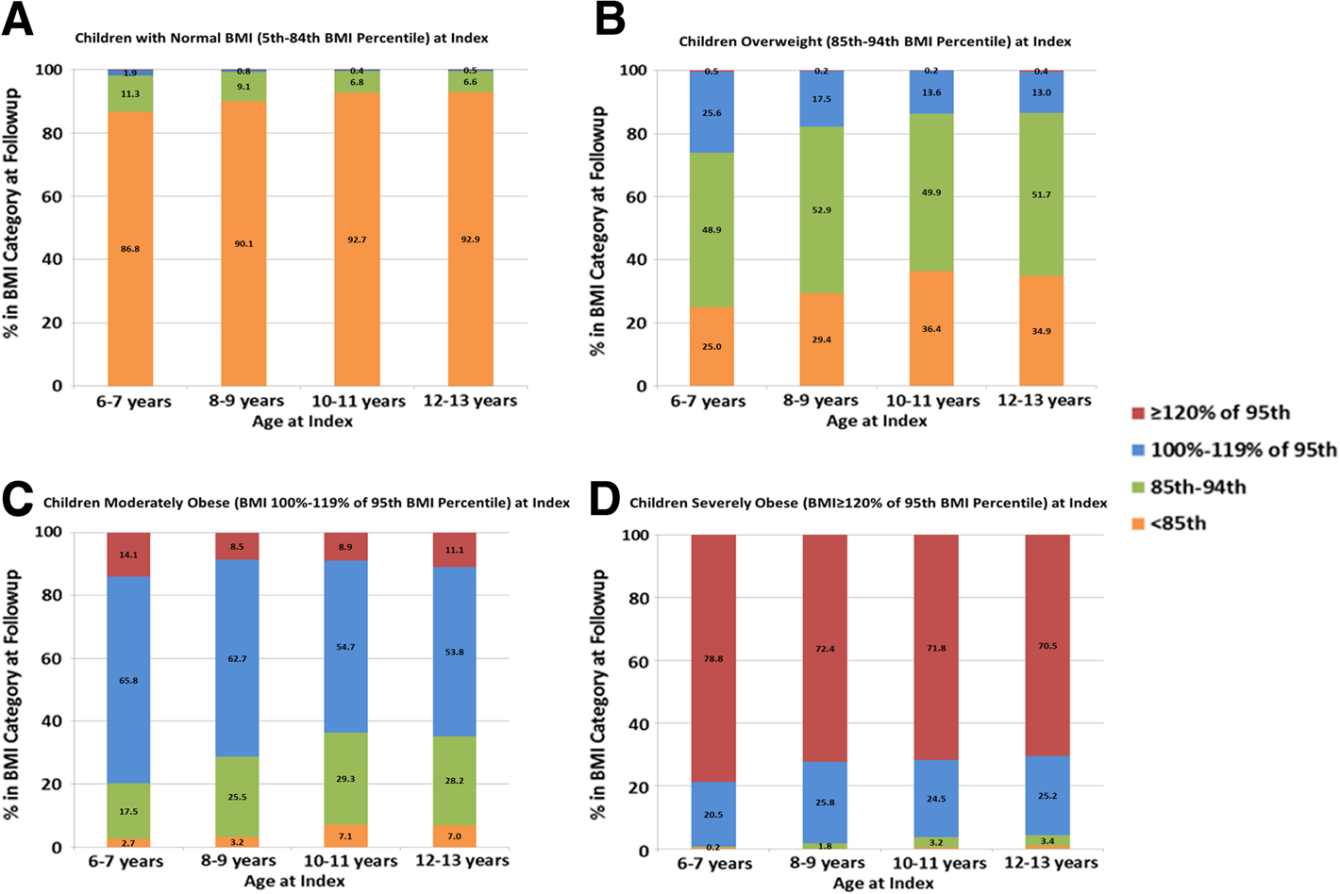
**Follow-up BMI category based on BMI percentile and BMI percentage of the 95**^**th **^**percentile by age and BMI at the index visit. (A)** For children with normal BMI at the index visit (N = 50,685), an increasing proportion of younger children were overweight at 2–3 years of follow-up (p < 0.001 comparing 6–7 and 8–9 year olds to 12–13 year olds, with no differences between 10–11 and 12–13 year olds, p = 0.6). **(B)** For children who were overweight at the index visit (N = 14,057), an increasing proportion of younger children were obese at follow-up (p <0.001, test for trend). **(C)** For children who were moderately obese at the index visit (BMI 100-119% of the 95^th^ percentile, N = 9848), an increasing proportion of younger children were obese at follow-up (p <0.001, test for trend). The proportions of moderately obese children who remained obese at follow-up were significantly different between all age groups (p <0.01) except for 10–11 year olds compared to 12–13 year olds. **(D)** For children who were severely obese at the index visit (N = 4295), nearly all were obese or severely obese at follow-up (95.7-99.3%). Children in the youngest age group (6–7 years old) had the highest prevalence of severe obesity at follow-up compared to other age groups (p <0.05). Differences between the older age groups were not significantly different.

There was a strong tendency for obese children to remain in an obese BMI category over the follow-up period of this study. Follow-up analyses conducted using data from 80,697 children age 6–13 years old at the index visit and 2–3 years older at the time of subsequent BMI measurement (median 2.3 years, interquartile range 2.1-2.8 years after the index visit) demonstrated that 71.9% of children with severe obesity continued to be severely obese (with an even greater proportion among younger children) and an additional 24.7% remained obese but their BMI declined to the moderately obese range (Figure 2D). Among moderately obese children, 10.5% became severely obese (14.1% for children ages 6–7) while 57.1% remained moderately obese and 26.6% became overweight but not obese (Figure 2C). Less than one fifth (15.3%) of overweight children became moderately obese, while half (51.2%) remained overweight and one third (33.2%) had follow-up BMI in the normal range (Figure 2B).

Among obese children, we also examined whether there was a graded relationship between increasing BMI percentage above the 95^th^ percentile and elevated blood pressure. As shown in Table 3, the proportion of children with normal blood pressure fell while those with prehypertension or hypertension increased as BMI percentage increased above the 95^th^ percentile. Adjusting for differences in age, sex and race/ethnicity, a significant graded relationship between obesity severity and hypertension remained, with the adjusted odds of hypertension increasing across increasing BMI strata: OR 1.5 (CI 0.9-2.3) for BMI 110-119%, OR 1.8 (CI 1.1-3.0) for BMI 120-129%, OR 3.5 (CI 2.1-5.8) for BMI 130-139%, and OR 5.7 (CI 3.6-8.9) for BMI ≥140% of the 95^th^ BMI percentile, compared to BMI 100-109% of the 95^th^ BMI percentile. Severe obesity (BMI ≥120% of the 95^th^ BMI percentile) was associated with a 2.7-fold increased odds of hypertension compared to those with moderate obesity (BMI 100-119% of the 95^th^ percentile; adjusted OR 2.7, CI 2.0-3.7). These results were similar when restricting analyses to the subset of 11,705 obese children and adolescents who had three consecutive BP measurements (data not shown).

**Table 3:** **Blood pressure in obese children and adolescents classified based on percentage of the 95**^**th **^**percentile for body mass index (BMI)**

**BMI > 95**^**th **^**percentile**	**Normal blood pressure (BP)**	**Prehypertension**	**BP ≥95**^**th **^percentile^*^	**Confirmed hypertension**	**Adjusted odds of hypertension**
	**N = 12802**	**N = 5308**	**N = 2796**	**N = 166**	**OR (95% CI)**^**†**^
100-109% × 95^th^ percentile	5976 (67.2%)	2012 (22.6%)	903 (10.2%)	40 (0.5%)	-
110-119% × 95^th^ percentile	3478 (62.9%)	1390 (25.2%)	659 (11.9%)	36 (0.7%)	1.5 (0.9 – 2.3)
120-129% × 95^th^ percentile	1789 (56.8%)	866 (27.5%)	497 (15.8%)	24 (0.8%)	1.8 (1.1 – 3.0)
130-139% × 95^th^ percentile	837 (49.5%)	515 (30.5%)	339 (20.1%)	26 (1.5%)	3.5 (2.1 – 5.8)
≥140% × 95^th^ percentile	722 (43.9%)	525 (31.9%)	398 (24.2%)	40 (2.4%)	5.7 (3.6 – 8.9)

row percentages.

^*^For those with an index BP ≥95^th^ percentile, up to 3 consecutive measurements were used to assign final BP status. Hypertension was defined by 3 consecutive BP ≥95^th^ percentile (or SBP ≥140 mmHg and/or DBP ≥90 mmHg for subsequent BP at age ≥18 years of age). Within this subgroup, there were 1900 (67.9%) children and adolescents with repeat BP <95^th^ percentile and 730 (26.1%) with no subsequent BP available for final assignment.

^†^Odds ratio (OR) and 95% confidence intervals (CI), adjusted for differences in age, sex and race/ethnicity.

## Discussion

This study extends results from previously published studies of pediatric obesity by examining gradations in severe obesity by gender, age and race/ethnicity. In addition, we evaluated the persistence of obesity and obesity severity over 2–3 years, as well as the association between obesity severity and hypertension. Severe obesity was found in 5.6% of children and adolescents age 6–17 years old, with prevalence highest among children of Hispanic ethnicity or black race, similar to findings for overall obesity (BMI ≥95^th^ percentile). National data [7] and other racially and ethnically diverse cohorts [12] also demonstrate a disproportionately higher prevalence of severe obesity in Hispanic boys and non-Hispanic black girls. Furthermore, longitudinal data on BMI tracking from more than 80,000 children and adolescents included within this study cohort (of whom 9848 were moderately obese and 4295 severely obese at the index visit) demonstrate that severely obese children tend to maintain the same degree of obesity over time, particularly when evident at a young age. These findings support data from other longitudinal pediatric cohorts documenting a strong tracking effect of elevated BMI in childhood, as well as into adulthood [18-23]. Similar shifts in BMI across normal, overweight, obese and severely obese categories were also observed in a multisite school-based study of 3993 U.S. sixth graders re-examined later in eigth grade, where 76% of severely obese youths (alternatively defined by the 99^th^ BMI percentile) remained severely obese after 2.5 years of follow-up [24].

While previous findings from this source cohort demonstrated that pediatric obesity was significantly related to an increased prevalence of hypertension [13], in this study we applied new obesity stratification criteria and found that the risk of hypertension increased as the degree of obesity increased, with a nearly three-fold greater risk of hypertension among severely obese children compared to moderately obese children. These findings are consistent with the known interrelationship of adiposity, hypertension and cardiometabolic factors in children [5,25-29], supporting evidence of greater risk with extreme or severe obesity [5,30-32], and further quantify the association between obesity severity and hypertension as a representation of cardiovascular health risk. The persistence of severe obesity in longitudinal analyses, supporting data from other pediatric studies examining longitudinal shifts in BMI category among smaller population cohorts [24], also documents an ongoing highly significant adverse cardiovascular impact and emphasizes the need for early weight loss management.

These data have several clinical and public health implications. First, consistent with recently published concensus statements emphasizing the importance of identifying and tracking severe obesity in children and adolescents [10], current approaches to classifying obesity in the clinical setting should be refined at the higher end of the BMI spectrum to more precisely identify those with severe obesity. Categorization based strictly on BMI percentiles does not adequately stratify risk within the extreme BMI range (>99^th^ percentile). In addition, conventional growth charts have a BMI limit of 36–37 kg/m^2^, which is problematic for tracking of weight status in adolescents with extremely high BMI [11]. In this study, we found that classifying obesity severity based on BMI percentage of the 95^th^ percentile, as previously proposed by others [9,11], has the advantage of more precisely categorizing extremely obese individuals for both clinical tracking and research purposes. More importantly, our data demonstrate that this new obesity classification system results in clinically relevant thresholds of higher order obesity associated with graded health risk, as defined by hypertension.

Several factors may constrain the interpretation of our data. First, these data were limited to a subset of children receiving well-child care in a northern California healthcare delivery system and thus may not be fully representative of other population demographics or regions. Second, there is the theoretical possibility that growth chart reference data may differ by age, with implications for examining longitudinal trends. However, our BMI thresholds focus on well-established reference data for the 85^th^ and 95^th^ BMI percentiles thresholds, deriving higher order obesity based on the 95^th^ percentile rather than the 97^th^ or 99^th^ BMI percentiles. Third, while we believe the measurements of BP in these subjects were accurate, assessment of BP in extremely obese children is a challenge due to limitations in cuff size [26,33,34]. Finally, we limited our cardiovascular risk assessment to blood pressure, since lipid measurements were not uniformly available to further estimate cardiovascular risk in addition to blood pressure. As such, we believe that the previously documented tracking effect for blood pressure in this age range to adulthood [17] supports the use of hypertension as an important component of overall cardiovascular risk.

In summary, among the substantial number of children with obesity (BMI at or above the 95^th^ percentile), identifying subsets of those with more extreme obesity is of clinical importance. Stratification of obesity by percentage above the 95^th^ BMI percentile shows a graded increased risk of hypertension and may be related to increased risk of other cardiovascular risk factors such as dyslipidemia and prediabetes. A more refined and precise classification of severe pediatric obesity using contemporary criteria [10] may serve to improve clinical practice, tracking, as well as public health surveillance, and facilitate research on the best approaches to managing cardiovascular risk in these subgroups of varying obesity severity.

## Competing interests

The authors declare that they have no competing interests.

## Authors’ contributions

JL, SD, AS and LG conceptualized and designed the study. MC conducted the data analyses. JL, LG, BM, AS and RP drafted the initial manuscript. All authors (JL, MC, AS, SD, RP, BM, EP, NS, MD, EK, KA, DM, PO, LG) provided critical input on data analysis and interpretation, revised the manuscript for important intellectual content and approved the final version.
